# Endophthalmitis caused by *Shinella* species: the first case report

**DOI:** 10.1186/s12348-026-00610-0

**Published:** 2026-06-16

**Authors:** Qibin Xiao, Bin Chen, Zhike Xu, Zhiwei Cui

**Affiliations:** Department of Ophthalmology, People’s Hospital of Leshan, Leshan, Sichuan Province China

**Keywords:** *Shinella*, Endophthalmitis, Pars plana vitrectomy, Ocular infection, Metagenomic next-generation sequencing (mNGS)

## Abstract

**Background:**

Endophthalmitis is a severe intraocular infection associated with potentially devastating visual outcomes. *Shinella*, a Gram-negative bacillus commonly found in water and soil, has never been reported as a cause of human disease.

**Case presentation:**

A 49-year-old female farmer presented with a 7-day history of vision loss, ocular irritation, and ophthalmalgia in her right eye. She had been previously misdiagnosed and treated with high-dose systemic corticosteroids at another institution. She underwent emergent pars plana vitrectomy. Vitreous samples were analyzed using conventional culture and metagenomic next-generation sequencing (mNGS), which identified Shinella species as the predominant pathogen. Intravitreal amikacin and systemic ceftazidime were initiated on postoperative day 3 after culture confirmed Gram-negative bacilli. Two weeks of targeted antibiotic therapy resulted in complete resolution of intraocular inflammation and near-full visual recovery.

**Conclusion:**

To our knowledge, this is the first reported case of intraocular infection caused by Shinella species. This case highlights Shinella as a potential ocular pathogen and demonstrates the utility of pars plana vitrectomy combined with mNGS for diagnosing atypical intraocular infections.

## Introduction

*Shinella* is a Gram-negative, non-spore-forming, rod-shaped genus belonging to the family Rhizobiaceae, first described in 2006 [1]. Nine species are validly recognized, predominantly isolated from wastewater sludge, contaminated soil, and other environmental matrices [2]. Despite its environmental prevalence, *Shinella* spp. have no established pathogenicity in humans, and no ocular infections have been previously reported [3]. We describe the first case of *Shinella*-associated endophthalmitis, highlighting the value of pars plana vitrectomy and molecular diagnostic techniques in identifying rare and atypical pathogens.

## Case presentation

**Fig. 1:**
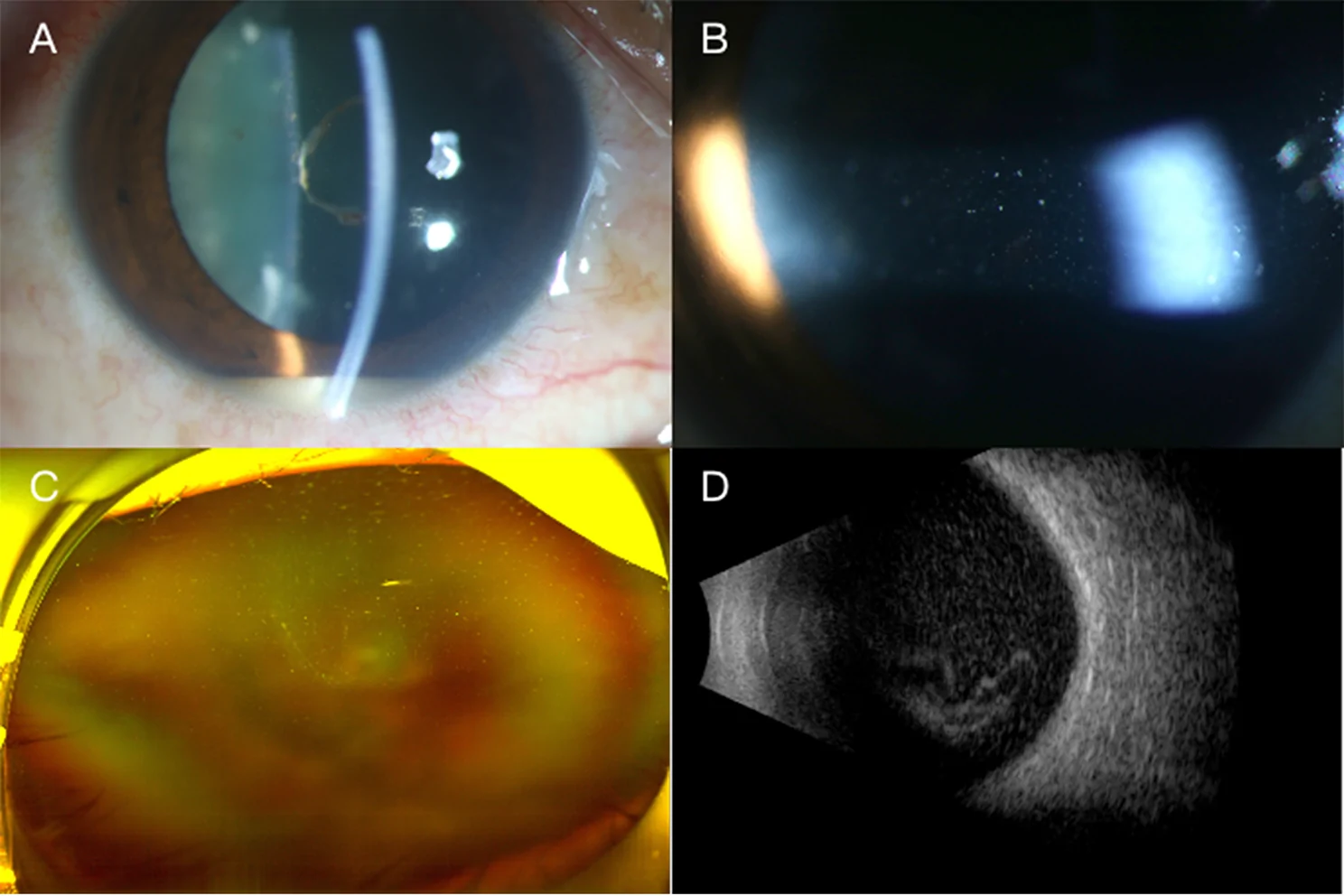
(**A**, **B**) Anterior segment photographs of the right eye show conjunctival hyperemia, a transparent cornea with fine keratic precipitates, a deep anterior chamber with turbid aqueous humor and hypopyon. A Vossius ring is visible. (**C**) Color fundus photograph shows an invisible fundus due to dense vitritis. (**D**) B-scan ultrasonography demonstrates diffuse vitreous opacities

A 49-year-old female presented with a 7-day history of vision loss, ocular irritation, and pain in her right eye. According to examination reports from another hospital, her initial visual acuity was hand motion with accurate projection in the right eye (OD) and 20/25 in the left eye (OS). Intraocular pressure was 15 mmHg OD and 20 mmHg OS. Slit-lamp biomicroscopy revealed fine keratic precipitates, 2 + anterior chamber cells, posterior synechiae, and 3 + vitreous cells in the right eye; the fundus was not visible (Fig. 1). The left eye was unremarkable. The patient, a farmer by occupation, reported regular contact with soil and wastewater during family fishpond maintenance. She denied any history of ocular trauma, and no ocular wounds were identified on detailed examination. She had received high-dose systemic corticosteroids at the previous hospital; no systemic or intravitreal antibiotics were administered there. Despite corticosteroid therapy, her visual acuity failed to improve.

**Fig. 2:**
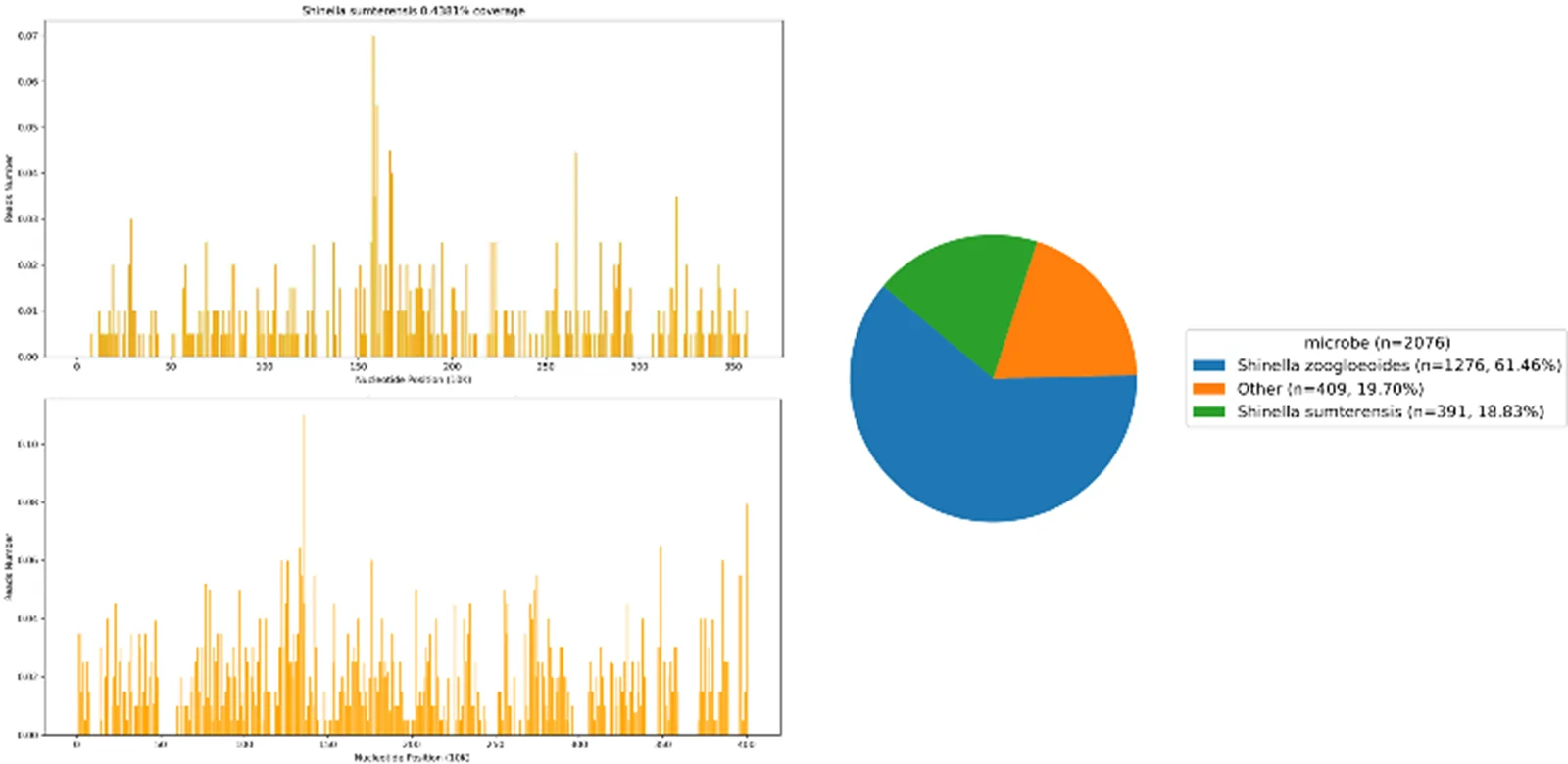
Metagenomic next-generation sequencing analysis of vitreous fluid, revealing *Shinella* nucleotide reads as the predominant sequence

**Fig. 3:**
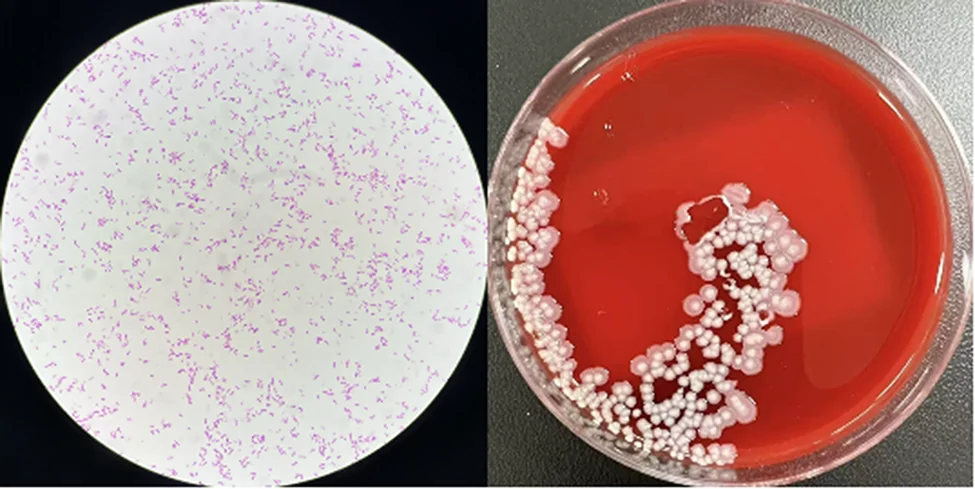
Conventional bacterial culture demonstrating growth of Gram-negative bacilli from the vitreous sample

She was admitted with a preliminary diagnosis of panuveitis. Systemic corticosteroids were immediately discontinued because infectious endophthalmitis could not be excluded. No systemic corticosteroids were used thereafter. A comprehensive laboratory workup—including complete blood count, erythrocyte sedimentation rate, rheumatoid factor, antinuclear antibodies, and serological tests for tuberculosis, human immunodeficiency virus, syphilis, and hepatitis—was unremarkable. B-scan ultrasonography of the right eye showed dense vitreous opacities without retinal detachment. Given the unclear etiology of intraocular inflammation, emergent pars plana vitrectomy was performed. Vitreous samples were obtained for conventional cultures and sent to Beijing Genetron Medical Diagnostics Laboratory for mNGS and cytokine profiling. The mNGS identified *Shinella* species (Fig. 2), and cytokine profiling indicated marked intraocular inflammation. Conventional cultures confirmed Gram-negative bacilli on postoperative day 3 (Fig. 3).

Preoperatively and immediately postoperatively, no systemic or intravitreal antibiotics were administered. Intravitreal amikacin injections (0.4 mg/0.1 mL, twice weekly) and intravenous ceftazidime (2 g every 12 h) were initiated on postoperative day 3, after culture results confirmed Gram-negative bacilli. Topical levofloxacin (four times daily), tobramycin/dexamethasone eye drops (four times daily) and ointment (once nightly), and tropicamide (twice daily) were continued locally. Systemic ceftazidime was discontinued after one week, while intravitreal amikacin was continued for an additional week. During this period, supplementary workup including blood cultures, abdominal ultrasound, chest X-ray, and cranial computed tomography showed no abnormalities.

**Fig. 4:**
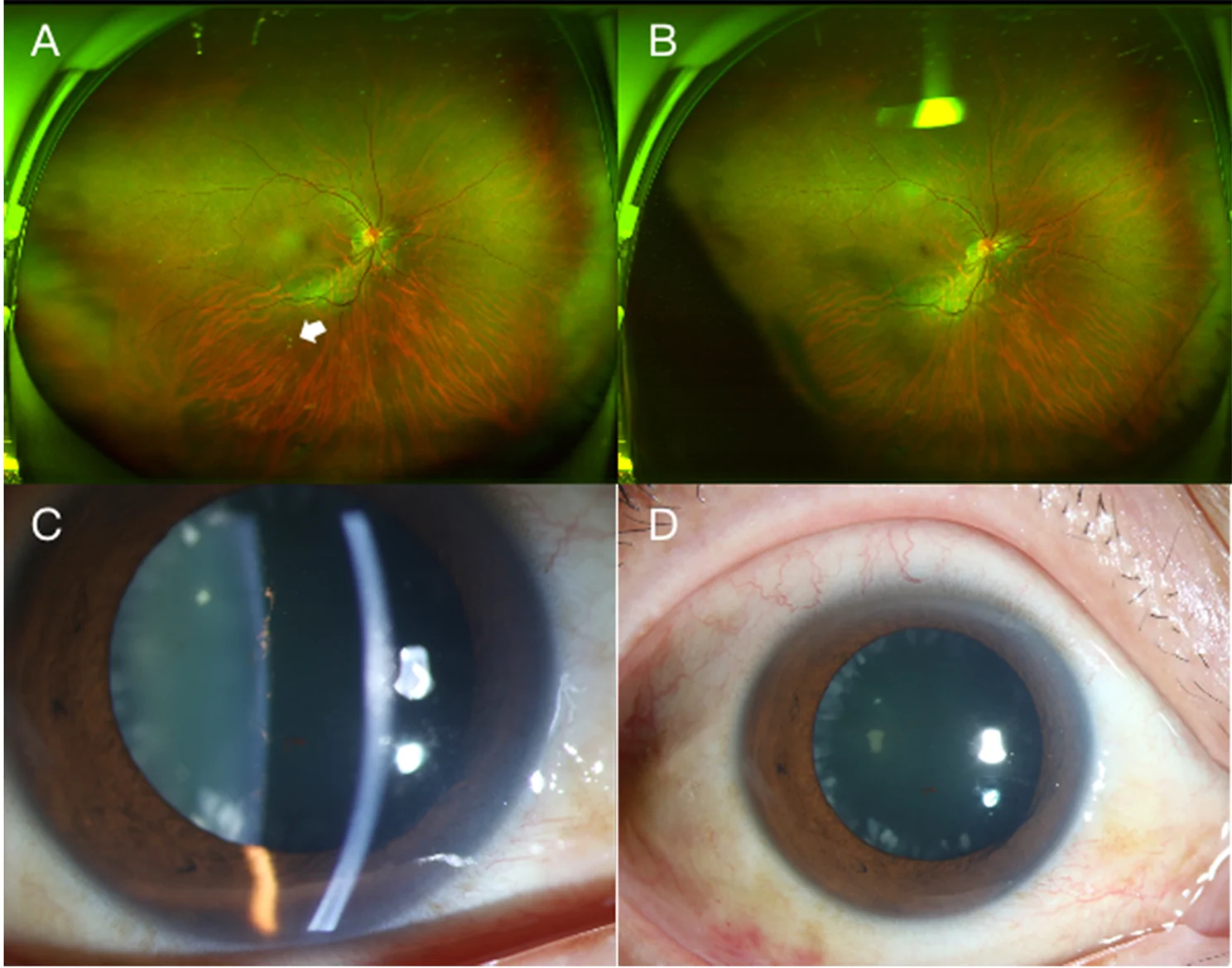
Post-treatment clinical course of the right eye. (**A**) Color fundus photograph at one week showing resolution of vitritis; an inflammatory aggregate (presumed bacterial) is visible inferotemporal to the vascular arcade (arrow). (**B**) At two weeks, the aggregate has resolved with normal fundus appearance. (**C**) Anterior segment photograph at one week showing marked reduction in conjunctival hyperemia and anterior chamber inflammation. (**D**) At two weeks, the anterior chamber is quiet with a clear cornea

After one week of targeted antibiotic therapy, visual acuity improved to 20/200 (OD), further increased to 20/30 (OD) after two weeks. Ocular examination revealed a clear cornea, quiet anterior chamber, clear vitreous cavity, and a normal retina. The patient was followed up for 6 months after resolution of the endophthalmitis, with no signs of recurrent intraocular inflammation (Fig. 4).

## Discussion

*Shinella* is a genus of Gram-negative, rod-shaped, motile bacteria belonging to the family Rhizobiaceae, which includes well-known nitrogen-fixing genera Rhizobium and Bradyrhizobium [1]. First proposed in 2006, *Shinella* species were previously classified under the genus Rhizobium and named in honor of Korean microbiologist Young-Kee Shin for his contributions to rhizobial research. These bacteria are commonly recovered from soil, water, and environmental sludge [2, 3]. Historically regarded as an environmental organism with no established human pathogenicity, *Shinella* has not been linked to intraocular disease prior to this report [4–6].

The patient’s occupation as a farmer poses a risk of frequent exposure to soil and wastewater, a key environmental risk factor for contact with *Shinella* [5]. She denied any history of ocular trauma, and no ocular wounds were identified on detailed examination; however, an exogenous infection route cannot be excluded. This includes unrecognized corneal or conjunctival microtrauma during farm work, or occult cutaneous wounds at other body sites leading to transient bacteremia and subsequent hematogenous seeding to the eye. Routine blood tests, including blood cultures, often fail to detect such transient, low-grade bacteremia. Given the absence of a definitive systemic infectious source, an endogenous route cannot be confirmed; the possibility of exogenous infection remains plausible and cannot be ruled out.

Antimicrobial management followed a precise timeline guided by clinical uncertainty and subsequent microbiological confirmation: no systemic or intravitreal antibiotics were administered preoperatively or immediately postoperatively, because the clinical course was atypical, and an infectious etiology could not be confirmed upfront. Targeted therapy with intravitreal amikacin and systemic ceftazidime was initiated on postoperative day 3, once conventional culture confirmed Gram-negative bacilli [6]. Antimicrobial susceptibility testing was not performed due to resource limitations, but the regimen was guided directly by microbiological results.

Clinically, *Shinella*-associated endophthalmitis presents with non-specific signs indistinguishable from other bacterial endophthalmitis: ocular pain, conjunctival hyperemia, and rapid vision loss [7, 8]. This patient’s 1-week acute course and favorable visual prognosis different from the severe, often devastating outcomes of other Gram-negative bacillary endophthalmitis, though this single case observation cannot be generalized [8]. Larger case series are needed to clarify *Shinella*’s pathogenicity, clinical spectrum, and optimal treatment strategies.

Notably, high-dose systemic corticosteroids administered at the previous institution temporarily relieved symptoms and partially reduced visible inflammation but failed to control the underlying intraocular infection. The patient’s vision did not improve despite apparent symptomatic relief, highlighting the risk of immunosuppressive therapy masking infectious endophthalmitis [8]. On admission to our hospital, systemic corticosteroids were immediately discontinued because infectious endophthalmitis could not be excluded. No systemic corticosteroids were used at any point during our management; only topical tobramycin/dexamethasone eye drops and ointment were applied locally to control persistent inflammation.

In this case, mNGS confirmed *Shinella* directly from vitreous fluid. Strict sterile sampling and laboratory protocols were followed to minimize contamination, but low-level environmental contamination cannot be entirely excluded, given that *Shinella* is commonly found in water and soil. Importantly, mNGS results were fully concordant with conventional culture findings, validating its reliability for identifying atypical or rare pathogens that may be missed by routine culture [9].

Pars plana vitrectomy played a critical, multi-faceted therapeutic role in this case. First, it directly removed the infected, inflamed vitreous gel, which serves as a nutrient-rich reservoir for bacterial proliferation; reducing the bacterial load significantly mitigated intraocular inflammation and prevented progressive retinal and choroidal damage. Second, vitrectomy provided sterile, uncontaminated vitreous specimens for conventional culture and mNGS testing, enabling definitive identification of the rare *Shinella* pathogen—an essential step for guiding targeted antimicrobial therapy. Third, it cleared dense vitreous opacities that obscured the fundus, facilitating postoperative monitoring of retinal status and treatment response. Without emergent pars plana vitrectomy, both diagnostic accuracy and therapeutic outcomes would have been severely compromised [10].

This case has key limitations. First, antimicrobial susceptibility testing was not performed, so the in vitro activity of amikacin and ceftazidime against *Shinella* remains unconfirmed. Second, the exact infection route is unclear, with an exogenous pathway remaining plausible and not definitively excluded. Third, environmental or sampling contamination cannot be fully ruled out, despite strict sterile procedures.

## Conclusion

To our knowledge, this is the first documented case of *Shinella*-associated endophthalmitis, identifying *Shinella* as a novel potential ocular pathogen. In patients with environmental exposure (e.g., farmers) and unexplained endophthalmitis, an exogenous infection route cannot be excluded. For unclear intraocular inflammation, diagnostic pars plana vitrectomy combined with mNGS is critical for identifying rare or atypical pathogens. Systemic corticosteroids should be strictly withheld when infectious endophthalmitis cannot be excluded, as immunosuppression may exacerbate underlying infection. Further studies are needed to clarify *Shinella*’s pathogenic mechanisms, exact transmission pathways, and antimicrobial susceptibility to guide clinical practice.

## Data Availability

No datasets were generated or analysed during the current study.

## Abbreviations

OD: Right eye
OS: Left eye
mNGS: Metagenomic next-generation sequencing
